# Thoracic Excursion Is a Biomarker for Evaluating Respiratory Function in Amyotrophic Lateral Sclerosis

**DOI:** 10.3389/fneur.2022.853469

**Published:** 2022-03-23

**Authors:** Naohiko Iguchi, Tomoo Mano, Naoki Iwasa, Maki Ozaki, Nanami Yamada, Naoya Kikutsuji, Akira Kido, Kazuma Sugie

**Affiliations:** ^1^Department of Neurology, Nara Medical University, Kashihara, Japan; ^2^Department of Rehabilitation Medicine, Nara Medical University, Kashihara, Japan

**Keywords:** amyotrophic lateral sclerosis, biomarker, pulmonary function test, respiratory function, thoracic excursion

## Abstract

**Objective:**

To evaluate the usefulness of thoracic excursion as a biomarker in patients with amyotrophic lateral sclerosis (ALS).

**Methods:**

We measured the forced the vital capacity (FVC), thoracic excursion, baseline-to-peak diaphragmatic compound muscle action potential (DCMAP) amplitude, diaphragm thickness at full inspiration (DTfi), Medical Research Council (MRC) sum score for muscle strength, and arterial partial pressures of oxygen and carbon dioxide and administered the Amyotrophic Lateral Sclerosis Functional Rating Scale-Revised (ALSFRS-R) and modified Medical Research Council (mMRC) Dyspnea Scale. The test–retest reliability of thoracic excursion was determined.

**Results and Conclusions:**

Thirty-four patients with ALS and 26 age- and sex-matched healthy participants were enrolled. Thoracic excursion measurement had excellent test–retest reliability (intraclass coefficient: 0.974). Thoracic excursion was more strongly correlated with FVC (*r* = 0.678, *p* < 0.001) than DCMAP amplitude (*r* = 0.501, *p* = 0.003) and DTfi (*r* = 0.597, *p* < 0.001). It was also correlated with ALSFRS-R score (*r* = 0.610, *p* < 0.001), MRC sum score (*r* = 0.470, *p* = 0.005), and mMRC Dyspnea Scale score (*r* = −0.446, *p* = 0.008) and was the most sensitive parameter for assessing dyspnea and FVC. Thoracic excursion decreased as FVC declined in the early and late stages, there were no differences in DCMAP amplitude and DTfi between the early and late stages, and ALSFRS-R score and MRC sum score decreased only in the late stage. Thoracic excursion was well correlated with respiratory function and is useful for predicting respiratory and general dysfunction in patients with ALS regardless of stage.

## Introduction

Amyotrophic lateral sclerosis (ALS) is a neurodegenerative disorder in which respiratory dysfunction occurs with disease progression. Eventually, the diaphragm and other muscles of respiration are involved, resulting in respiratory muscle paralysis, which is the primary cause of death. Respiratory function is most frequently assessed using the pulmonary function test (PFT). PFT parameters, such as forced vital capacity (FVC) and forced expiratory volume in 1 s, have been found to predict hypoventilation (1, 2) and survival (3–5) in patients with ALS. However, PFT has been associated with a risk of viral infection, and PFT results may be inaccurate due to air leakage from around the breathing tube and through the glottis because of facial muscle weakness, especially in patients with bulbar onset ALS (6–8). Alternative methods for measuring respiratory function and predicting FVC based on neurophysiological principles have recently been reported. FVC has been found to be correlated with diaphragmatic compound muscle action potential (DCMAP) elicited through phrenic nerve stimulation (9–11) and diaphragm thickness (DT) measured using ultrasonography (12, 13). However, these methods require a high level of skill. Thoracic excursion is estimated by measuring chest expansion. It is known to correlate with maximal inspiratory volume and is used as an outcome in respiratory rehabilitation. We aimed to determine the usefulness of thoracic excursion as an alternative to PFT in patients with ALS.

## Materials and Methods

### Ethical Considerations

This study was approved by the Ethics Committee of Nara Medical University (approval #2688). All study procedures were performed in accordance with the ethical standards of the ethics committee, Declaration of Helsinki, and Ethical Guidelines for Medical and Health Research Involving Human Subjects in Japan (UMIN: 000042222). All participants provided written and verbal informed consent after receiving information about the study.

### Study Population

We included all patients diagnosed with definite, probable, or possible ALS based on the modified El Escorial criteria (14) who visited Nara Medical University Hospital between June 2020 and October 2021. The inclusion criteria were as follows: (1) patients aged between 20 and 90 years; (2) patients without tracheostomy; and (3) patients not on non-invasive positive pressure ventilation. The exclusion criteria were as follows: (1) patients who received drugs for ALS that had not been approved in Japan or had undergone surgery in the previous 12 months (48 weeks); (2) patients with dementia (Mini-mental State Exam score <10); (3) patients with severe psychiatric disorders; (4) patients who were unable to answer questionnaires; (5) patients with suicidal ideation; (6) patients undergoing treatment for respiratory disease; (7) patients with severe heart failure; (8) patients who were pregnant; and (9) patients judged to be inappropriate for this study by the investigator or coordinator.

We selected control participants from the in- and out-patients of the Departments of Neurology at Nara Medical University Hospital, Nara and Minami Nara General Medical Center, Nara. We excluded control participants with subjective breathlessness and those who could not walk.

### General Assessment

The participants were administered the Amyotrophic Lateral Sclerosis Functional Rating Scale-Revised (ALSFRS-R). Total ALSFRS-R scores range from 0 to 48, and low scores strongly predict a poor outcome. In addition, ALSFRS-R consists of three subscales, which were scored separately: bulbar function (three items: speech, salivation, and swallowing), extremities function (six items: handwriting, cutting food and handling utensils, dressing and hygiene, turning in bed and adjusting bed clothing, walking, and climbing stairs), and respiratory function (three items: dyspnoea, orthopnoea and respiratory insufficiency). We used a cutoff score of 38 because patients with an ALSFRS-score <38 have a high 1-year mortality rate (15). Additionally, we graded the muscle strength of the limbs using the Medical Research Council (MRC) sum score, an established multifactorial scoring system with a total score between 0 and 60 (16). The MRC sum score evaluates global muscle strength by combining the scores of six muscles (shoulder abductors, elbow flexors, wrist extensors, hip flexors, knee extensors, and ankle dorsiflexors) that are evaluated bilaterally. We used an MRC sum score cutoff of 51, as an MRC sum score <51 has been reported to be associated with a poor outcome and high risk of mechanical ventilation in patients with neuromuscular disease (17). For assessment of dyspnea, the participants were administered the Modified Medical Research Council (mMRC) Dyspnea Scale. Patients who were unable to walk and those with dyspnea during activities of daily living and caregiving in bed were given a score of 4 and those with no dyspnea were given a score of 0.

### Thoracic Excursion Measurement

**Figure 1:**
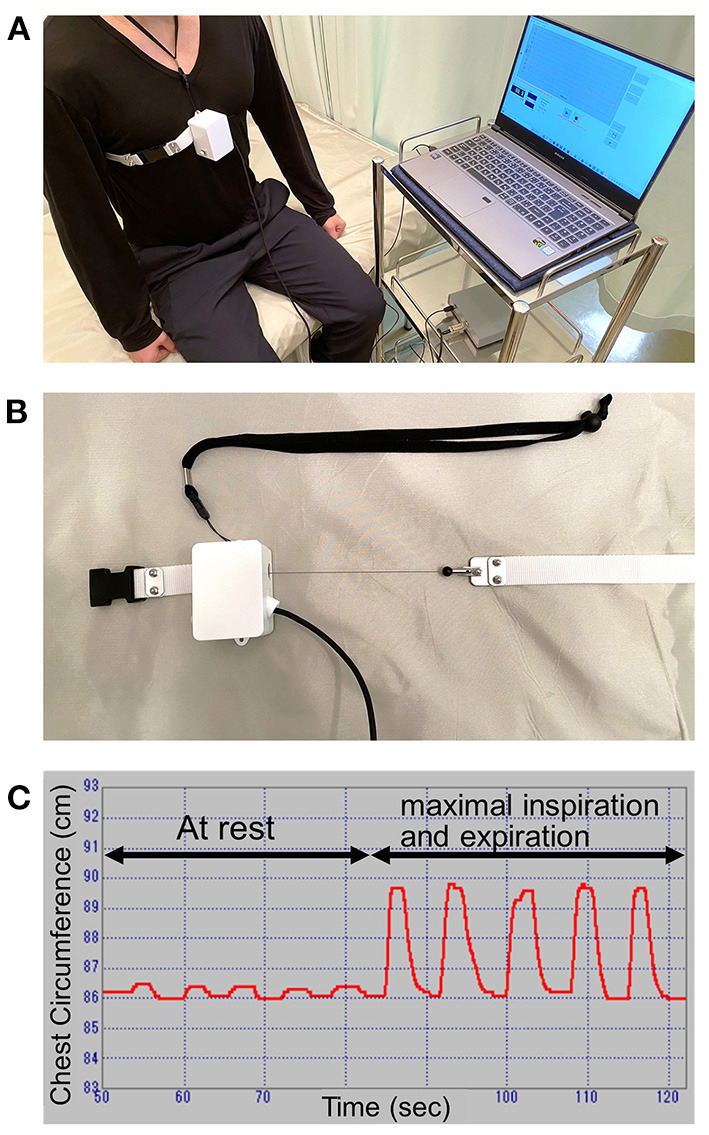
Thoracic excursion measurement. **(A)** The scenery of thoracic excursion measurement. **(B)** The wire of the measurement device. **(C)** The display of the application.

Thoracic excursion from maximal inspiration to maximal expiration was measured using a measurement system (Takeikikikougyo, Niigata, Japan) that continuously measured chest expansion and automatically recorded the data. The measurement of thoracic excursion had high intra- and inter-rater reliability and validity (18). A wire was placed over the xiphoid process and horizontally wrapped around the trunk (Figures 1A,B). Thoracic excursion was measured during five timed maximal inspirations and expirations, and the average of the five measurements was used for analysis (Figure 1C).

### Phrenic Nerve Conduction Study

We performed electrical phrenic nerve stimulation at the neck to elicit a DCMAP, which was recorded using surface electrodes. The active electrode was placed over the xiphoid process, and the reference electrode was placed at the costal margin 16 cm away from the active electrode (9). The phrenic nerve was supra-maximally stimulated three times on each side in all participants. The response with the highest baseline-to-peak amplitude was selected on each side, and the average amplitude of both sides was used for analysis.

### Ultrasonography

DT was measured between the anterior and mid-axillary lines with the participant in the supine position using a Logiq S8 ultrasonography machine (GE Healthcare, Chicago, IL, USA) with a high-resolution 3–12 MHz linear probe in the B mode (19, 20). The diaphragm was identified as a relatively non-echogenic muscle layer surrounded by two echogenic layers that represented the diaphragmatic pleural and peritoneal membranes (21, 22). DT was measured at three points bilaterally at the end of inspiration, and the average diaphragmatic thickness at full inspiration (DTfi) of both sides was used for analysis.

### PFT

PFT was performed with the participants seated upright. Spirometry was used to test pulmonary mechanics through the measurement of the percent-predicted FVC, forced expiratory volume in 1 s, forced expiratory and inspiratory flow rates, and maximal voluntary ventilation. These values are used to assess the ability of the lungs to quickly move large volumes of air through the airways and to identify airway obstruction. To identify the parameter that is most involved in the decline in FVC, we divided the participants into the severe (<50% FVC; late stage of FVC decline), mild (50– <80%; early stage of FVC decline), and no restriction (≥80%) groups. The two patients who were not able to perform PFT because of difficulty in breathing were included in the severe group.

### Statistical Analysis

Based on the assumption that the difference in thoracic excursion between the groups would be 0.8 cm with a standard deviation of 1.0 cm, we set a target sample size of 26 participants in each group (ALS group and control group) to ensure that the power is at least 80% with a two-sided significance level of 0.05. Intraclass coefficients were calculated to determine the test-retest reliability of the measurement of thoracic excursion; both “test” and “re-test” were performed by the same examiner. The Shapiro–Wilk test was used to assess the distribution of the data, and Pearson's correlation coefficients (*r*) were calculated to evaluate the associations between thoracic excursion and respiratory function. Based on the *r* values, the correlation was categorized as weak (*r* = 0.30–0.39), moderate (*r* = 0.40–0.59), or strong (*r* = 0.60–1.00). We used the *t*-test to compare the two groups and the Tukey–Kramer *post-hoc* test for multiple comparisons. Statistical significance was set at *p* < 0.05. Statistical analyses were performed using SPSS version 22.0 (SPSS Japan, Tokyo, Japan).

## Results

### Clinical Characteristics and Thoracic Excursion of Patients With ALS and Control Participants

**Figure 2:**
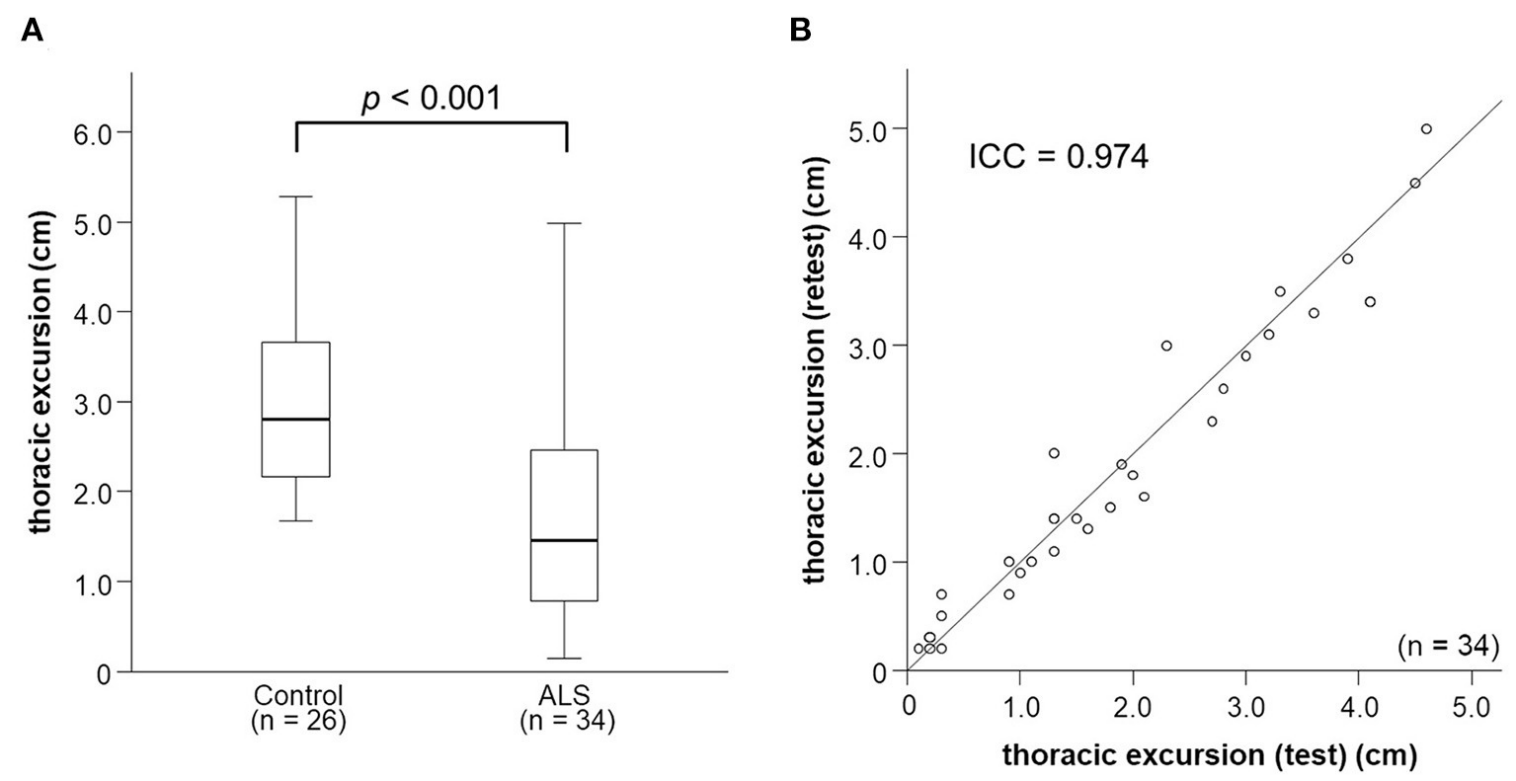
Thoracic excursion in patients with ALS. **(A)** The values of thoracic excursion in patients with ALS are significantly smaller than those in control patients. **(B)** Thoracic excursion measurement shows excellent test-retest reliability. ALS, amyotrophic lateral sclerosis.

We enrolled 34 consecutive patients with sporadic ALS and 26 control participants. Their clinical characteristics are presented in Table 1. In patients with ALS, the mean age was 70.1 ± 11.5 years (range: 39–88) and the mean disease duration was 22.4 ± 22.0 months. Twenty-six patients had spinal-onset ALS, and the remaining eight patients had bulbar-onset ALS. Between the two groups, there was no difference in FVC and neurophysiological parameters. ALSFRS-R bulbar function score and age at examination were lower in bulbar-onset ALS patients than spinal-onset ALS patients (Table 2). In addition, there was no difference between the patients with ALS and control participants in sex, age at examination, body mass index, and smoking history, although thoracic excursion was significantly worse in the patients with ALS (Figure 2A).

**Table 1:** Demographic characteristics of patients with ALS and control participants.

**Clinical features**	**ALS (***n*** = 34)**	**Control (***n*** =26)**	***p*** **-value**
Gender (female)	13 (38.2%)	10 (38.5%)	0.986
Onset symptoms	8/26	N.A.	
Bulbar/Spinal			
Age at examination (years)	70.1 ± 11.5 (39–88)	69.5 ± 11.1 (45–88)	0.835
Age at onset (years)	68.4 ± 11.8 (39–87)	N.A.	
Disease duration (months)	22.4 ± 22.0 (3–90)	N.A.	
BMI (kg/m^2^)	21.0 ± 4.1 (14.8–34.1)	21.6 ± 3.5 (15.8–29.5)	0.544
Past and current smoking	17 (50.0%)	14 (53.8%)	0.772
mMRC Dyspnea Scale score	2.0 ± 1.6 (0–4)	0.0 ± 0.0 (0)	<0.001
Thoracic excursion (cm)	1.72 ± 1.30 (0.14–4.98)	2.93 ± 0.92 (1.68–5.28)	<0.001
DCMAP amplitude (mV)	0.36 ± 0.23 (0.044–1.025)	N.A.	
DTfl (mm)	2.03 ± 0.86 (0.743–3.823)	N.A.	
FVC (*n* = 32)	75.1 ± 28.0 (24.7–125.1)	N.A.	
PaCO_2_ (mmHg)	42.1 ± 5.76 (32.1–60.1)	N.A.	
PaO_2_ (mmHg)	88.9 ± 14.0 (66.6–129.0)	N.A.	
ALSFRS-R score	33.7 ± 8.05 (18–48)	N.A.	
MRC sum score	46.2 ± 12.0 (8–60)	N.A.	

*Data are shown as mean ± SD (range) and number (percentage). ALS, amyotrophic lateral sclerosis; N.A., not applicable; BMI, body mass index; mMRC, modified Medical Research Council; DCMAP, diaphragmatic compound motor-action potential; DTfl, diaphragm thickness at full inspiration; FVC, forced vital capacity; PaCO_2_, arterial partial pressures of carbon dioxide; PaO_2_, arterial partial pressures of oxygen; ALSFRS-R, amyotrophic lateral sclerosis functional rating scale-revised; MRC, medical research council*.

**Table 2:** Demographic characteristics of patients with spinal-onset and bulbar-onset ALS.

**Onset type**	**Spinal-onset (***n*** = 26)**	**Bulbar-onset (***n*** = 8)**	***p*** **-value**
Gender (female)	9 (34.6%)	4 (50.0%)	0.679
Age at examination (years)	67.9 ± 11.7 (39–85)	77.3 ± 7.6 (67–88)	<0.05
Age at onset (years)	65.9 ± 11.9 (39–85)	76.5 ± 7.2 (67–86)	<0.05
Disease duration (months)	25.9 ± 23.9 (3–90)	11.0 ± 6.9 (5–26)	0.096
BMI (kg/m^2^)	21.2 ± 4.5 (14.8–34.1)	20.6 ± 2.4 (16.8–24.4)	0.720
mMRC Dyspnea Scale score	2.3 ± 1.6 (0–4)	1.1 ± 1.5 (0–4)	0.076
Thoracic excursion (cm)	1.73 ± 1.41 (0.14–4.98)	1.70 ± 0.88 (0.54–3.32)	0.943
DCMAP amplitude (mV)	0.35 ± 0.25 (0.044–1.025)	0.41 ± 0.14 (0.143–0.632)	0.516
DTfl (mm)	2.08 ± 0.85 (0.743–3.277)	1.88 ± 0.92 (0.963–3.823)	0.556
FVC	75.7 ± 30.4 (24.7–125.1) (*n* = 25)	72.9 ± 18.6 (50.2–100.9) (*n* = 7)	0.820
PaCO_2_ (mmHg)	42.8 ± 6.4 (32.1–60.1)	39.9 ± 2.1 (36.7–43.0)	0.214
PaO_2_ (mmHg)	88.6 ± 14.1 (66.7–129.0)	89.6 ± 14.7 (66.6–107.0)	0.874
ALSFRS-R score	32.5 ± 8.1 (18–48)	37.9 ± 6.9 (26–46)	0.097
ALSFRS-R bulbar function score	10.1 ± 2.3 (5–12)	6.4 ± 2.8 (2–10)	<0.01
ALSFRS-R extremities function score	11.9 ± 6.4 (0–24)	19.6 ± 5.1 (12–24)	<0.01
ALSFRS-R respiratory function score	10.7 ± 1.4 (6–12)	11.9 ± 0.4 (11–12)	<0.05
MRC sum score	43.5 ± 12.3 (8–60)	55.0 ± 4.6 (46–60)	<0.05

*Data are shown as mean ± SD (range) and number (percentage). ALS, amyotrophic lateral sclerosis; BMI, body mass index; mMRC, modified Medical Research Council; DCMAP, diaphragmatic compound motor-action potential; DTfl, diaphragm thickness at full inspiration; FVC, forced vital capacity; PaCO_2_, arterial partial pressures of carbon dioxide; PaO_2_, arterial partial pressures of oxygen; ALSFRS-R, amyotrophic lateral sclerosis functional rating scale-revised; MRC, medical research council*.

### Test–Retest Reliability of Measurement of Thoracic Excursion

The intraclass coefficient of thoracic excursion was 0.974 (Figure 2B), which indicates excellent test–retest reliability.

### Relationships Among Thoracic Excursion, Other Neurophysiological Parameters, and Clinical Characteristics

**Figure 3:**
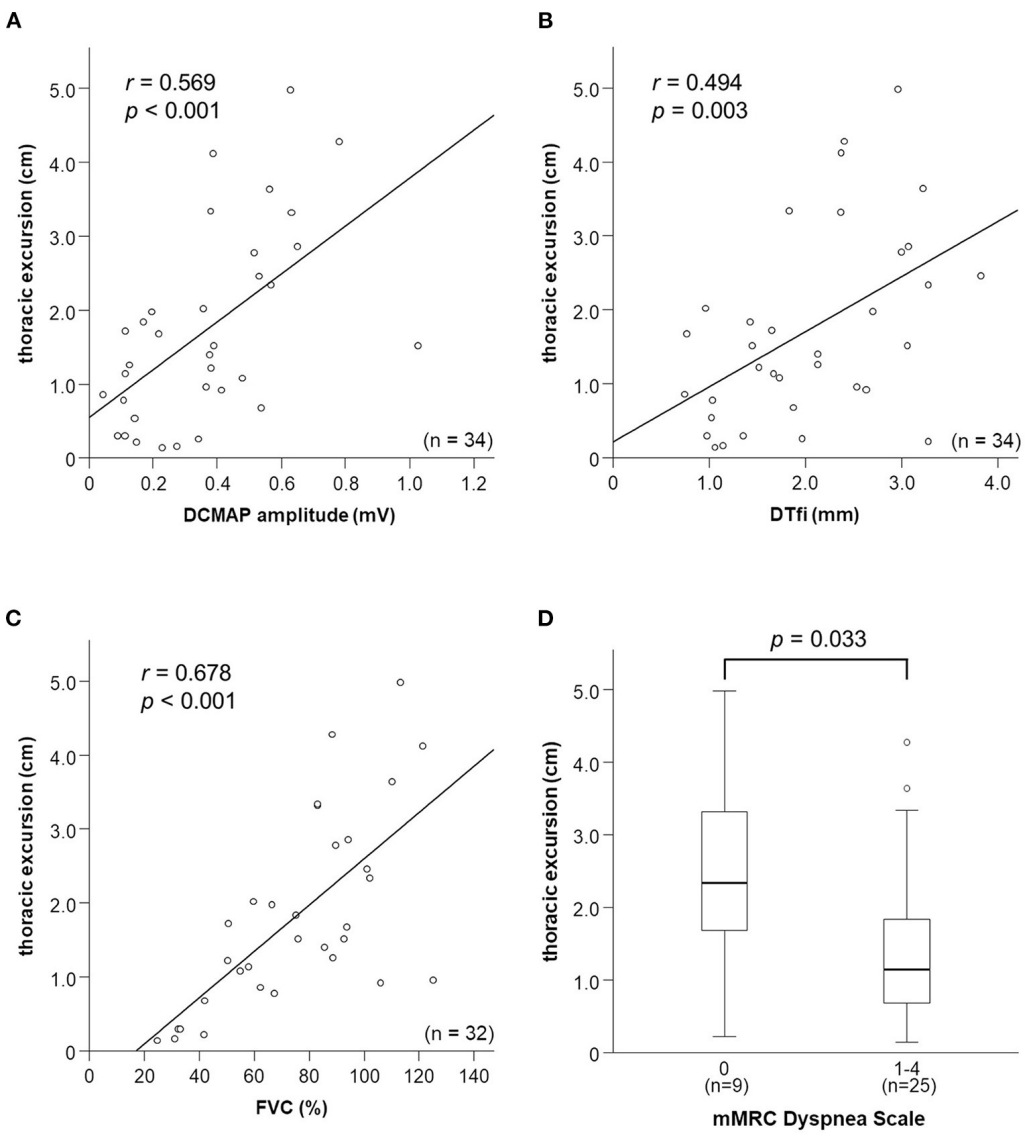
Relationships between thoracic excursion and other neurophysiological parameters, FVC, and mMRC Dyspnea Scale score. **(A–C)** Relationships between thoracic excursion and DCMAP amplitude, DTfi, and FVC. Thoracic excursion shows a strong correlation with FVC **(C)** and moderate correlation with DCMAP amplitude **(A)** and DTfi **(B)**. **(D)** Relationships between thoracic excursion and subjective dyspnea severity assessed using the mMRC Dyspnea Scale. Thoracic excursion is the most sensitive parameter for assessing subjective dyspnea. FVC, forced vital capacity; mMRC, modified Medical Research Council; DCMAP, diaphragmatic compound muscle action potential; DTfi, diaphragm thickness at full inspiration.

Thoracic excursion was moderately correlated with DCMAP amplitude (*r* = 0.569, *p* < 0.001) and DTfi (*r* = 0.494, *p* = 0.003; Figures 3A,B). DCMAP amplitude was strongly correlated with DTfi (*r* = 0.621, *p* < 0.001). Thoracic excursion was strongly correlated with FVC (*r* = 0.678, *p* < 0.001; Figure 3C), whereas DCMAP amplitude (*r* = 0.501, *p* = 0.003) and DTfi (*r* = 0.597, *p* < 0.001) were only moderately correlated with FVC. Conversely, there were no correlations between thoracic excursion and the patients' clinical characteristics, such as age at examination (*r* = −0.163, *p* = 0.358), age at onset (*r* = −0.113, *p* = 0.524), disease duration (*r* = −0.258, *p* = 0.141), and body mass index (*r* = −0.228, *p* = 0.195). Likewise, thoracic excursion was not correlated with age at examination (*r* = −0.143, *p* = 0.487) and body mass index (*r* = −0.055, *p* = 0.788) in control participants.

In addition, we investigated the relationship between mMRC Dyspnea Scale score, neurophysiological parameters, and FVC. mMRC Dyspnea Scale score was moderately correlated with thoracic excursion (*r* = −0.446, *p* = 0.008) and FVC (*r* = −0.428, *p* = 0.015) and weakly correlated with DCMAP amplitude (*r* = −0.318, *p* = 0.067) and DTfi (*r* = −0.392, *p* = 0.022). Patients with an mMRC Dyspnea Scale score of 1–4 had a significantly lower thoracic excursion than those with a score of 0 (Figure 3D). There were no differences in other parameters, such as FVC, DCMAP amplitude, and DTfi, between the mild and severe respiratory dysfunction groups (Supplementary Figures 1A–C).

### Relationships Between Neurophysiological Parameters and ALSFRS-R Score

**Figure 4:**
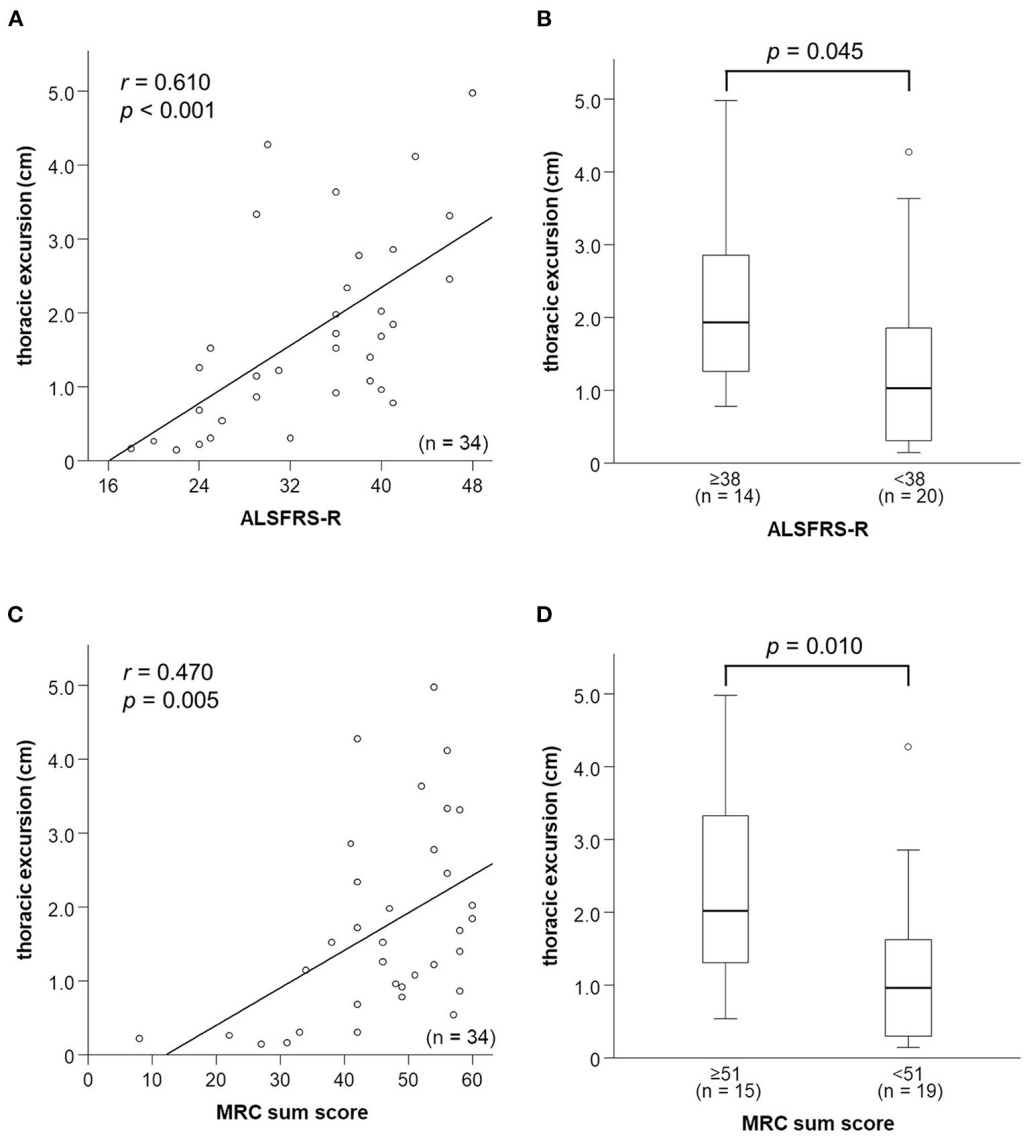
Relationships between respiratory function parameters and general condition. **(A)** Relationship between thoracic excursion and ALSFRS-R score. Thoracic excursion is strongly correlated with ALSFRS-R score. **(B)** Thoracic excursion is significantly lower in the patients in the mild respiratory dysfunction group whose ALSFRS-R score is <38 than those whose ALSFRS-R score is not <38. **(C)** Relationship between thoracic excursion and MRC sum score. Thoracic excursion is strongly correlated with MRC sum score. **(D)** Thoracic excursion is significantly lower in patients in the mild respiratory dysfunction group whose MRC sum score is <51 than in those whose MRC sum score is not <51. ALSFRS-R, amyotrophic lateral sclerosis functional rating scale-revised; MRC, medical research council.

ALSFRS-R score was strongly correlated with thoracic excursion (*r* = 0.610, *p* < 0.001; Figure 4A) and FVC (*r* = 0.622, *p* < 0.001). In contrast, DTfi was weakly correlated with ALSFRS-R score (*r* = 0.308, *p* = 0.076), and DCMAP amplitude was not correlated with the ALSFRS-R score (*r* = 0.239, *p* = 0.173; Supplementary Figures 2A–C). Furthermore, patients with an ALSFRS-R score < 38 had a significantly lower thoracic excursion and FVC than those with an ALSFRS-R score of 38 or more (Figure 4B). In contrast, there were no differences in DCMAP amplitude and DTfi between the mild and severe respiratory dysfunction groups (Supplementary Figures 2D–F). In ALSFRS-R sub-scores analysis, FVC correlated with both ALSFRS-R extremities (*r* = 0.500, *p* = 0.004) and bulbar function score (*r* = 0.370, *p* = 0.037). Contrary to this, thoracic excursion correlated only with ALSFRS-R extremities function score (*r* = 0.584, *p* < 0.001), not with bulbar function score (*r* = 0.227, *p* = 0.197). Additionally, in bulbar-onset ALS patients, FVC correlated strongly with ALSFRS-R bulbar function score (*r* = 0.709, *p* = 0.075) and moderately with extremities function score (*r* = 0.506, *p* = 0.247). In contrast, FVC correlated strongly with ALSFRS-R extremities function score (*r* = 0.678, *p* < 0.001) and weakly with bulbar function score (*r* = 0.367, *p* = 0.072) in spinal-onset ALS patients. Similarly, thoracic excursion was strongly correlated with ALSFRS-R extremities function score in both spinal-onset (*r* = 0.676, *p* < 0.001) and bulbar-onset ALS (*r* = 0.696, *p* = 0.055).

### Relationships Between Neurophysiological Parameters and Limb Muscle Weakness

We examined the relationship between neurophysiological parameters, FVC, and MRC sum score. MRC sum score was moderately correlated with thoracic excursion (*r* = 0.506, *p* = 0.006; Figure 4C) and FVC (*r* = 0.564, *p* = 0.003; Supplementary Figure 3A). In contrast, it was not correlated with DCMAP amplitude and DTfi (Supplementary Figures 3B,C). Patients with a low (<51) MRC sum score had a significantly lower thoracic excursion and FVC than those with a high MRC sum score (Figure 4D). In contrast, there were no differences in DCMAP amplitude and DTfi between the high and low MRC sum score groups (Supplementary Figures 3D–F). Therefore, limb muscle weakness was associated with a decline of thoracic excursion and FVC but not with diaphragmatic function.

### Relationships Between FVC and Neurophysiological Parameters

**Figure 5:**
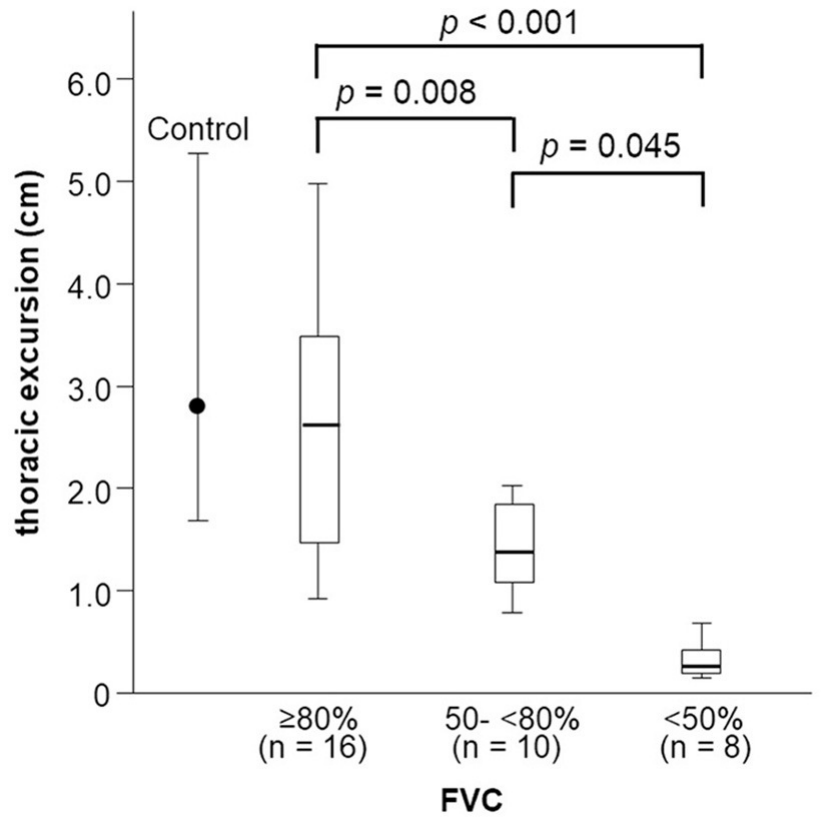
Changes in thoracic excursion with FVC decline. Thoracic excursion decreases in both the early and late stage of FVC decline in patients with ALS. FVC, forced vital capacity; ALS, amyotrophic lateral sclerosis.

**Figure 6:**
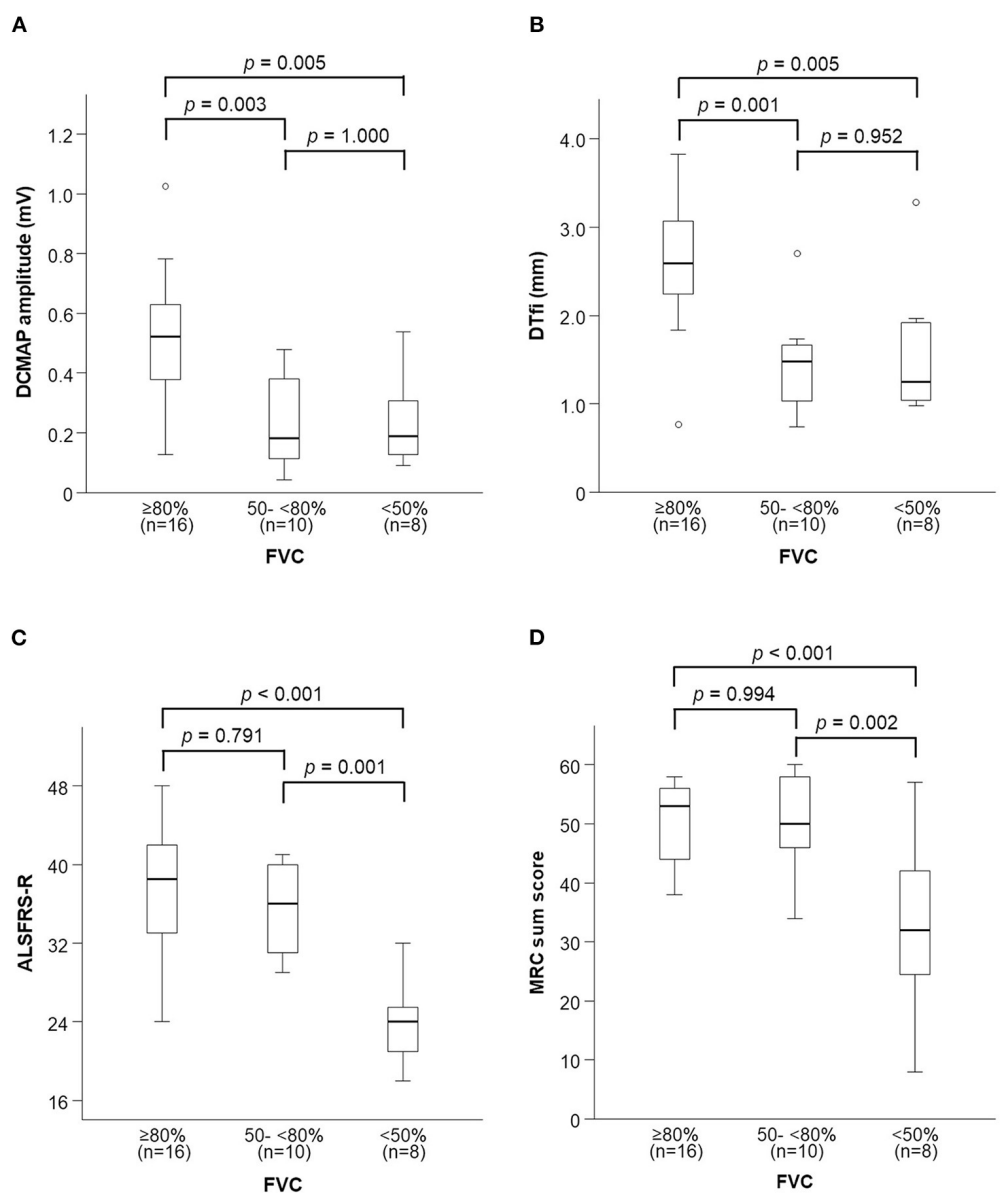
Changes in neurophysiological parameters and general condition with FVC decline. **(A,B)** DCMAP amplitude and DTfi decrease only in the early stage of FVC decline. **(C,D)** ALSFRS-R score and MRC sum score decrease only in the late stage of FVC decline. FVC, forced vital capacity; DCMAP, diaphragmatic compound motor-action potential; DTfl, diaphragm thickness at full inspiration; ALSFRS-R, amyotrophic lateral sclerosis functional rating scale-revised; MRC, medical research council.

Thoracic excursion decreased in both the early and late stages of FVC decline (Figure 5), but DCMAP amplitude and DTfi did not decrease in the late stage of FVC decline (Figures 6A,B). In contrast, ALSFRS-R score and MRC sum score did not decrease in the early stage of FVC decline, but they decreased in the late stage of FVC decline (Figures 6C,D).

## Discussion

This is the first study that used thoracic excursion measurement to evaluate respiratory dysfunction in patients with ALS. We found that thoracic excursion is a reliable and valid biomarker of respiratory function in patients with ALS. We observed that thoracic excursion was strongly correlated with FVC and can be used as a surrogate marker of FVC. Since FVC decline predicts hypoventilation (1, 2) and survival (3–5), a decline in thoracic excursion might also be able to predict hypoventilation and survival in patients with ALS. Furthermore, thoracic excursion is easier to measure than DCMAP amplitude and DT; it can be measured at home or in a clinic.

Thoracic excursion was a better predictor of respiratory dysfunction and general condition than DCMAP amplitude and DTfi. Phrenic nerve conduction study is a technique used for measuring respiratory function (9, 10). A previous study reported that the DCMAP amplitude of healthy individuals is 1.0 mV (10). DCMAP amplitude has been reported to be correlated with FVC and other respiratory parameters, such as maximum inspiratory pressure and sniff nasal inspiratory pressure (11), and it may be an indicator of prognosis in ALS (23–25). Low DCMAP amplitude has been found to predict a poor outcome in patients with ALS (11, 26). Diaphragm ultrasonography is another useful tool for measuring respiratory function (27, 28). DT at full expiration and full inspiration is correlated with FVC (12, 13, 21), as is DT ratio (21, 29). In patients with ALS, a low DT at expiration indicates atrophy of the diaphragm and hypoventilation, and a low DT at inspiration or low DT ratio indicates respiratory muscle weakness.

Thoracic excursion has been reported to correlate with inspiratory capacity (18). Previous studies have used chest expansion to assess the severity of rheumatologic diseases (30, 31) and the effects of inspiratory muscle training in patients with chronic obstructive pulmonary disease (32), ankylosing spondylitis (33), and myasthenia gravis (34). In patients with ALS, thoracic and abdominal excursion recorded using respiratory magnetometry were used to assess the response to diaphragm training (35). However, measurement of chest expansion had never been used for assessing pulmonary function in patients with ALS.

Thoracic excursion was strongly correlated with MRC sum score and ALSFRS-R score, and especially with ALSFRS-R extremities function score. The measurement of thoracic excursion involves the evaluation of ribcage movement and the function of all the muscles of respiration. Hence, respiratory function is affected by the strength of the muscles of the extremities, general condition, and movement of the diaphragm. In this report, we confirmed that the general condition and the strength of the muscles of the extremities strongly affect respiratory function in patients with ALS.

We found that decline in thoracic excursion was strongly correlated with the severity of respiratory function. There were no differences in DCMAP amplitude and DTfi between the mild and severe respiratory dysfunction groups, and ALSFRS-R score and MRC sum score decreased only in the severe group. These results suggest that the factors affecting respiratory function differ between the early- and late-stages of ALS. Diaphragm weakness may affect thoracic excursion and FVC in the early stage, and weakness of the limb muscles and accessory muscles of respiration may affect thoracic excursion and FVC in the late stage. During inspiration, contraction of the diaphragm expands the abdominal ribcage, and contraction of the intercostal muscles and accessory muscles of respiration simultaneously expands the pulmonary ribcage (36). In patients with diaphragmatic dysfunction, the intercostal muscles and accessory muscles of respiration expand the pulmonary ribcage to compensate for diaphragm failure during inspiration (37). As their condition progresses, patients with ALS with diaphragmatic dysfunction show paradoxical abdominal motion as inspiration primarily relies on the action of the intercostal muscles and accessory muscles of respiration and the diaphragm is drawn into the chest wall during inspiration because of its dysfunction (38). Additionally, increased compound muscle action potential amplitudes have been observed in the external intercostal muscles in an end-stage SOD1 transgenic rat model of ALS, which reflects compensation for decreased trans-diaphragmatic pressure due to diaphragm dysfunction (39). Therefore, when paradoxical abdominal motion is observed, DCMAP amplitude and DT findings suggestive of severe diaphragmatic dysfunction and decreased FVC and thoracic excursion can be expected. The intercostal muscles and other accessory muscles of respiration elevate and expand the thoracic rib cage and compensate for diaphragmatic dysfunction to limit the decrease in FVC and thoracic excursion. With the progression of ALS, weakness of these muscles appears with worsening general condition, leading to decreases in thoracic excursion and FVC. Therefore, evaluation of diaphragmatic dysfunction is adequate for assessing pulmonary function in the early stage of ALS. Notably, thoracic excursion is useful for the evaluation of pulmonary function and general condition in ALS regardless of stage. Among all the neurophysiological parameters and FVC, we found thoracic excursion to be the most sensitive for assessing dyspnea. Thoracic excursion predicts the appearance of subjective dyspnea and decline in objective respiratory dysfunction.

The results of clinical trials of disease-modifying therapies for neurodegenerative disorders underscore the need for testing before the onset of neurological symptoms and evaluating disease severity and progression regardless of stage (40–42). Although clinical trials of potential therapies have been done in ALS, definite efficacy has not been demonstrated in RCTs (43). These results appear to be partly attributable to the absence of established outcome measures as well as the limited number of patients, which may diminish statistical power. Our findings suggest that thoracic excursion could be used as a surrogate marker in clinical trials. In recent ALS clinical trials, the following are often used as endpoints: the change in the ALSFRS-R, the decrease of muscle strength and FVC, and time from the allocation day to the onset of an event (24-h use of non-invasive respiratory support, use of invasive respiratory support, or death) (42–45). ALSFRS-R is highly reproducible and objective, although there is a slight variation by therapy in short-term clinical trial. The time from the allocation day to an event needs to take into account the effects of other factors such as the patient's stage of inclusion and living environment (45). FVC is used to determine whether to provide respiratory support rather than the prediction of the prognosis, as it is difficult to reliably measure respiratory function even at an early stage in bulbar-onset ALS (46, 47). In this study, FVC had a weak correlation with bulbar function score in total ALS patients. Additionally, FVC correlated more strongly with bulbar function score than extremities function score in bulbar-onset ALS patients, but not in spinal-onset ALS patients. Bulbar palsy symptoms may affect the measurement of FVC, especially in bulbar-onset ALS patients. By contrast, thoracic excursion had no correlation with bulbar function score. Taken together, measurement of thoracic excursion is a useful evaluation method of respiratory function and may take the place of FVC as an endpoint of respiratory function in clinical trials, which is not affected by bulbar palsy symptoms.

The measurement of thoracic excursion is simple and non-invasive, does not involve a special technique and requires only chest binding, and can be performed even at the bedside. However, our procedure involves a few additional steps. Thoracic excursion must be measured in an upright sitting or standing position to avoid friction between the wire wound around the thorax and the backrest of the chair (in reclined sitting) or bed (in supine). The other limitation in this study is that thoracic excursion in excessively obese patients may not be measured accurately. The wire of the measurement system does not shrink easily because of subcutaneous fat. Additionally, in this study, we have attempted to tackle the major challenges associated with identifying a surrogate biomarker for evaluating respiratory function and prognosis in patients with ALS. However, floor and ceiling effects could not be adequately analyzed. A large-scale longitudinal study is necessary to confirm the results of this study.

## Data Availability Statement

The original contributions presented in the study are included in the article/Supplementary Material, further inquiries can be directed to the corresponding author/s.

## Ethics Statement

The studies involving human participants were reviewed and approved by Nara Medical University Hospital. The patients/participants provided their written informed consent to participate in this study.

## Author Contributions

NIg and TM: conceptualization and writing—original draft preparation. NIg, NIw, and NY: performed data acquisition and interpretation. MO: performed formal analysis. AK and KS: supervision. All authors have read and agreed to the published version of the manuscript.

## Funding

This work was supported by the Ministry of Education, Culture, Sports, Science, and Technology of Japan under grant KAKENHI No. 20K19453.

## Conflict of Interest

The authors declare that the research was conducted in the absence of any commercial or financial relationships that could be construed as a potential conflict of interest.

## Publisher's Note

All claims expressed in this article are solely those of the authors and do not necessarily represent those of their affiliated organizations, or those of the publisher, the editors and the reviewers. Any product that may be evaluated in this article, or claim that may be made by its manufacturer, is not guaranteed or endorsed by the publisher.

## Abbreviations

ALS: amyotrophic lateral sclerosis
FVC: forced the vital capacity
DCMAP: diaphragmatic compound muscle action potential
DTfi: diaphragm thickness at full inspiration
MRC: Medical Research Council
ALSFRS-R: Amyotrophic Lateral Sclerosis Functional Rating Scale-Revised
mMRC: modified Medical Research Council
PFT: pulmonary function test.
