# Supplementary material for: Thoracic Excursion Is a Biomarker for Evaluating Respiratory Function in Amyotrophic Lateral Sclerosis
Source: Front Neurol. 2022 Mar 23;13:853469. doi: 10.3389/fneur.2022.853469 (PMC8984343; doi:10.3389/fneur.2022.853469)
Supplement: Supplementary file 1 [file Data_Sheet_1.PDF]

Supplementary Material

1 Supplementary figure legends

Supplementary Figure 1. Relationships between neurophysiological parameters, FVC, and mMRC Dyspnea Scale score.

(A-C) There are no differences in FVC, DCMAP amplitude, and DTfi between patients with and without breathlessness.

FVC = forced vital capacity; mMRC = modified Medical Research Council; DCMAP = diaphragmatic compound motor-action potential; DTfl = diaphragm thickness at full inspiration

Supplementary Figure 2. Relationships between ALSFRS-R score and FVC, DCMAP amplitude, and DTfi.

(A-C) Correlation between ALSFRS-R score and pulmonary function parameters. FVC is strongly correlated with ALSFRS-R score (A). ALSFRS-R score shows a weak correlation with DTfi (C) and no correlation with DCMAP amplitude (B).

(D-F) FVC is significantly lower in patients in the severe respiratory dysfunction group whose ALSFRS-R score is less than 38 than in those whose ALSFRS-R is not less than 38 (D). There are no differences in DCMAP amplitude and DTfi between the two groups (E and F).

ALSFRS-R = Amyotrophic Lateral Sclerosis Functional Rating Scale-Revised; FVC = forced vital capacity; DCMAP = diaphragmatic compound motor-action potential; DTfl = diaphragm thickness at full inspiration

Supplementary Figure 3. Relationships between MRC sum score and FVC, DCMAP amplitude, and DTfi.

(A-C) Correlation between MRC sum score and pulmonary function parameters. FVC shows a moderate correlation with MRC sum score (A). DCMAP amplitude and DTfi show no correlation with MRC sum score (B and C).

(D-F) There are no differences in FVC, DCMAP amplitude, and DTfi between patients with a high MRC sum score and those with a low MRC sum score.

MRC = Medical Research Council; FVC = forced vital capacity; DCMAP = diaphragmatic compound motor-action potential; DTfl = diaphragm thickness at full inspiration
